# Feasibility Analysis of ECG-Based pH Estimation for Asphyxia Detection in Neonates

**DOI:** 10.3390/s24113357

**Published:** 2024-05-24

**Authors:** Nadia Muhammad Hussain, Bilal Amin, Barry James McDermott, Eoghan Dunne, Martin O’Halloran, Adnan Elahi

**Affiliations:** 1 Electrical and Electronic Engineering, University of Galway, H91 TK33 Galway, Ireland; 2 Translational Medical Device Lab, University of Galway, H91 TK33 Galway, Ireland; 3 School of Medicine, University of Galway, H91 TK33 Galway, Ireland

**Keywords:** asphyxiated neonates, arterial blood gas analysis, birth asphyxia, Dunn–Šidák post hoc test, ECG features, Kruskal–Wallis nonparametric test, Pan–Tompkins algorithm

## Abstract

Birth asphyxia is a potential cause of death that is also associated with acute and chronic morbidities. The traditional and immediate approach for monitoring birth asphyxia (i.e., arterial blood gas analysis) is highly invasive and intermittent. Additionally, alternative noninvasive approaches such as pulse oximeters can be problematic, due to the possibility of false and erroneous measurements. Therefore, further research is needed to explore alternative noninvasive and accurate monitoring methods for asphyxiated neonates. This study aims to investigate the prominent ECG features based on pH estimation that could potentially be used to explore the noninvasive, accurate, and continuous monitoring of asphyxiated neonates. The dataset used contained 274 segments of ECG and pH values recorded simultaneously. After preprocessing the data, principal component analysis and the Pan–Tompkins algorithm were used for each segment to determine the most significant ECG cycle and to compute the ECG features. Descriptive statistics were performed to describe the main properties of the processed dataset. A Kruskal–Wallis nonparametric test was then used to analyze differences between the asphyxiated and non-asphyxiated groups. Finally, a Dunn–Šidák post hoc test was used for individual comparison among the mean ranks of all groups. The findings of this study showed that ECG features (T/QRS, T Amplitude, Tslope, Tslope/T, Tslope/|T|, HR, QT, and QTc) based on pH estimation differed significantly (*p* < 0.05) in asphyxiated neonates. All these key ECG features were also found to be significantly different between the two groups.

## 1. Introduction

The neonatal period (the first 28 days of life) is a critical and vulnerable time for the newborn [1]. The World Health Organization (WHO) reported the global number of neonatal deaths as 2.3 million in 2022 [1]. Of these deaths, 75% occur in the first week of life, and approximately 42% (1 million) occur in the first 24 h [2]. The WHO has identified a lack of quality medical care and appropriate interventional treatments during the intrapartum period and early days of life as being key risk factors for poor outcomes. Of specific concern are intrapartum-related complications, particularly birth asphyxia, which is recognized as a primary cause of death [2].

Birth asphyxia is defined by the WHO as a “failure to initiate and sustain breathing at birth” [2]. Birth asphyxia is the leading cause of neonatal deaths, accounting for 23% of neonatal deaths globally [3]. The *American Journal of Obstetrics and Gynecology* (AJOBG) and the American Academy of Pediatrics give a more precise definition of birth asphyxia. According to this definition, a neonate is categorized as asphyxiated if the umbilical arterial pH is <7, the Apgar score is <4 for 5 min, and there is evidence of neurological symptoms and multiorgan dysfunction [4]. The most common causes of birth asphyxia are umbilical cord abnormalities (including umbilical cord compression and nuchal cord), uterine rupture during pregnancy or labor, placental abruption (premature detachment of the placenta from the uterus), placenta previa or low-lying placenta, and premature birth [5,6].

Birth asphyxia is a potential cause of neonatal death. However, short-term, and long-term morbidities are also associated with the condition. The immediate consequences of birth asphyxia may include hypoxia (lack of oxygen in tissues), hypercapnia (excess of carbon dioxide in the bloodstream), metabolic or respiratory acidosis, ischemia, and hypotension [7,8]. Neonates who survive this acute phase may develop long-term neurological sequelae including hypoxic-ischemic encephalopathy (HIE); cognitive impairment; speech and developmental delays; vision, hearing, and feeding impairments; emotional and behavioral disorders; and learning disabilities. About 25% of survivors of birth asphyxia develop prolonged neurodevelopment disorders including cognitive and motor impairment and cerebral palsy [9]. These sequelae are associated with poor treatment options and prognoses [5,10]. Traditionally, the primary and immediate modality used to assess birth asphyxia is arterial blood gas analysis in neonates. Assessing the birth asphyxia also includes Apgar scores and the manifestation of neurological disorders including sepsis and hypoxic-ischemic encephalopathy [11]. When monitored, birth asphyxia is managed by supplemental oxygen. In birth asphyxia, a low oxygen concentration (hypoxia) results in metabolic acidosis and tissue damage. However, a high concentration of oxygen (hyperoxia) resulting in metabolic alkalosis may also damage the tissues [12,13].

Blood gas analysis is an accurate monitoring method for assessing neonatal conditions after birth asphyxia. Blood gas analysis measures oxygenation and gas exchange acid-base disorder (metabolic acidosis or metabolic alkalosis). In blood gas analysis, pH is a crucial biomarker to evaluate the risk of metabolic acidosis and make the decision to admit neonates to the neonatal intensive care unit (NICU) [14]. In clinical settings, automated blood gas analyzers are frequently utilized and can deliver results in 10 to 15 min. However, there are concerns about the time delays including blood draw from arterial lines, on-scene time, and technical difficulties in using blood gas analyzers [15]. Also, the highly invasive and intermittent nature of monitoring blood gas analysis has emphasized the need for the noninvasive and continuous monitoring of oxygenation in neonates [16,17,18].

In clinical practice, pulse oximetry (PO) and transcutaneous oxygen monitoring (tcPO2) are used for noninvasive continuous oxygenation monitoring. PO is a quick, painless, and simple technique to monitor oxygenation in all patient groups, including neonates [19]. PO, a noninvasive technique, is extensively used in neonatal intensive care units to measure blood oxygen saturation. It offers real-time monitoring of the neonate’s oxygenation status, which is crucial in assessing asphyxia. However, its reliability can be affected by low perfusion states or neonatal movement. Additionally, the accuracy of PO (which is below 80%) is limited in determining the oxygen level in tissues [20]. Furthermore, pulse oximetry does not provide information about the metabolic changes occurring in the neonate or the specific cause of oxygen deprivation [21,22]. tcPO2 measures the partial pressure of oxygen through the skin. However, this test is influenced by the skin thickness, temperature, and how much contact gel is used, resulting in erroneous and false readings [17,22]. Additionally, PO and tcPO2 do not provide any information about the metabolic disorder, making blood gas analysis necessary to find metabolic anomalies in asphyxiated neonates [23].

In neonates who are experiencing asphyxia, it is also necessary to evaluate the cardiac dysfunction. Neonate cardiac dysfunction caused by birth asphyxia is one of the key factors affecting the severity of the disease [24]. Between 24% and 78% of birth asphyxia events can result in cardiac dysfunction. Ischemic cardiac injury lowers cardiovascular reserve and causes myocardial failure, hypotension, and bradycardia as clinical manifestations. These symptoms are associated with a higher risk of mortality and poor neurological outcomes [25]. A definitive diagnostic tool for hypoxic-ischemic myocardial injury is the electrocardiogram (ECG) [24]. Cardiac muscle contraction is accompanied by electrical changes known as depolarization, and the electrical activity of restoring the muscle to rest is referred to as repolarization, which is measured by an ECG signal. An ECG signal can be segmented into a P wave, which represents atrial depolarization, a QRS complex, which displays ventricular depolarization, and a T wave following the QRS complex, which indicates ventricular repolarization. Cardiac arrhythmias triggered by hypoxia, which result in changes in ECG signal, include abnormal electrical impulse formation, premature ventricular arrhythmias, sinus tachycardia, sinus bradycardia, and sinoatrial block. Increased HR shortens the ECG cycle, and all these cardiac arrhythmias are recognized by ECG characteristics [26,27,28]. Recent studies have shown that P-wave dispersion and corrected QT (QTc) dispersion are important markers for the diagnosis of arrhythmias in asphyxiated neonates [29,30,31]. While these studies primarily focused on the diagnosis of arrhythmias, other studies have investigated potential ECG features for the detection of hypoxia [32,33,34,35,36,37]. Notably, an experimental study in fetal sheep [34] demonstrated that the presence of delayed ST-waveform elevation is a significant indication of spontaneous hypoxia in the fetus experiencing pre-existing hypoxia. The metabolic disorders and physiological changes in ECG such as a change in T/QRS and ST-waveform are associated with severe hypoxia [34]. During hypoxia, myocardial glycogen reserves are quickly depleted leading to changes in ECG waveform such as an increase in the T-wave, a higher T/QRS ratio, and an elevated ST segment [32]. Moreover, a surge of catecholamines is released as a defensive mechanism in response to severe hypoxia, which shortens the QT and QTc [33]. Another study on the preterm ovine model [35] compared the waveform-based and interval-based ECG features. According to the results of this animal study, ECG features including interval-based HR and HR-corrected QT could be used to detect severe hypoxia in fetal sheep [35]. Martinek et al. [36] performed feature extraction and ECG analysis techniques to extract the fECG features including fetal heart rate (FHR) and heart rate variability (HRV), and performed ST-analysis to determine the fetal hypoxia [36]. While assessing the relationship between fetal ECG and fetal scalp-pH, A. Luttkus et al. reported that fetal ECG accurately detects intrapartum hypoxia and is similar to the results obtained from scalp-pH [37].

Most studies reported in the literature have mainly focused on determining fetal hypoxia, and there is limited work on ECG-based asphyxia characterization in neonates. The gold-standard clinical biomarker for the accurate diagnosis of asphyxia in neonates is pH, which is monitored through intermittent blood sampling and blood gas analysis. However, to the best of our knowledge, no study has previously investigated the relationship between blood pH and ECG in neonates. This investigation is of much clinical significance as pH is the best biomarker to determine neonatal metabolic acidosis and the most reliable marker to predict neonatal asphyxia. ECG-based characterization of neonatal asphyxia as an alternative to highly invasive blood sampling will enable continuous monitoring of neonates during the management of asphyxia.

This study aims to investigate the morphological ECG features that can be used to characterize pH value (i.e., whether the neonatal condition is normal or abnormal) in asphyxiated neonates. Such a capability in ECG would help the clinician in monitoring birth asphyxia noninvasively. In this study, a dataset containing 274 ECG segments recorded from 49 neonates, along with their simultaneously recorded pH values, was used to investigate the relationship between ECG features and pH values. After the preprocessing of data, principal component analysis and the Pan–Tompkins algorithm were used to compute the ECG features. The relationship between each ECG feature and pH was first evaluated using descriptive statistics, and then the Kruskal–Wallis nonparametric test was used to analyze the differences between the two groups (i.e., normal vs. abnormal pH). Finally, the Dunn–Šidák post hoc test was utilized for individual comparison among the mean ranks of all groups.

The rest of this paper is organized as follows: Section 2 presents the materials and methods used in this study; Section 3 describes the results and discussion; and finally, conclusions are drawn in Section 4.

## 2. Materials and Methods

### 2.1. Dataset and Selection Criteria

The dataset used in this study has also been used in previous studies [38,39] and is a private neonatal dataset. The dataset was collected from the Neonatal Intensive Care Unit (NICU) of the Cork University Maternity Hospital, Ireland, in 2014. The dataset is comprised of 49 neonates (under 28 days of age, varying from 1 to 25 days) undergoing clinically perinatal asphyxia. Demographic and clinical information (gender, gestational age, birth weight, mode of delivery, type of neonatal resuscitation, days in NICU, and neonatal outcome) was collected and recorded for this study. The written parental consent of neonates was obtained under the ethical approval of the clinical research committee of Cork Teaching Hospitals. The dataset consists of recorded ECG signals in the time domain with a sampling rate of 256 Hz together with blood gas sample collection for blood gas analysis. Blood samples were evaluated for oxygenation state and acid-base disorders including pH, partial pressure of carbon dioxide (paCO_2_), and bicarbonate (HCO_3_). The neonates in this study were recruited if they fulfilled two or more of the following criteria: initial capillary or arterial pH < 7.1; Apgar score < 5 at 5 min; and initial capillary or arterial lactate > 7 mmol/L. All recruited neonates were term infants (≥37 weeks of gestation). This is a unique dataset as the dataset contains raw ECG signals (ECG signals without any preprocessing steps) and simultaneous intermittent measurements of arterial pH using blood gas analysis. Therefore, the dataset allows for the evaluation of the potential of various ECG features to characterize pH noninvasively.

### 2.2. pH Threshold in Neonates

Umbilical cord blood analysis is used to determine the risk of asphyxia in neonates. Arterial blood gas analysis has been utilized to determine the threshold values of acid-base disorders in asphyxiated neonates [12]. The initial step in evaluating acid-base imbalance involves assessing the pH value. However, the threshold values for fetal pH cannot be used in neonates due to changes in gas exchange right after delivery [40]. Thorborg et al. [41] explained the threshold ABG values based on the age of the neonates for prebirth (pH > 7.20), at 5 min after birth (pH = 7.20–7.34), and 1–7 days after birth (pH = 7.35–7.45). According to Tan et al. [42], the ideal environment for cellular metabolism accepts a range between 7.30 and 7.45 in neonates. This is lower than in older children (pH = 7.38–7.42), and there is an alkalosis if pH > 7.45, while metabolic acidosis in neonates is considered if pH < 7.20 [43].

### 2.3. Signal Preprocessing and Extraction of ECG Features

The ECG electrodes were placed on the neonate in a standard 12-lead configuration. However, the data acquisition was performed using a two-channel recording setup, with the electrodes positioned on the right and left sides of the midline of the sternum, corresponding to lead V1 and V2, respectively. The ECG data are analyzed through a three-step methodology: segmentation, preprocessing, and feature extraction. Each ECG signal is segmented [44], followed by the application of standard filters to remove common artifacts [45,46,47,48]. QRS complex detection is performed using the PT algorithm due to its established efficacy in detecting QRS [49,50]. T-wave features are extracted through PCA and PT algorithms [49,50,51,52,53] to determine the relevant ECG characteristics used in this study. This methodology builds upon established practices in the field of ECG signal processing, leveraging well-established techniques to ensure accurate and reliable analysis.

**Figure 1:**
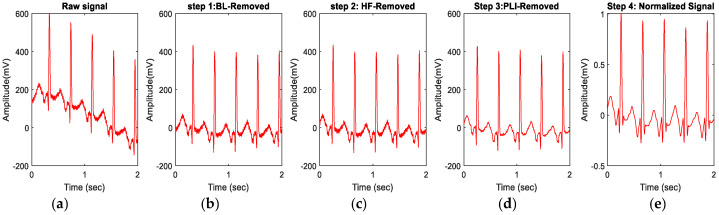
Preprocessing steps performed in this work: (**a**) Raw ECG signal; (**b**) BL-Removed: ECG signal after baseline (BL) noise removal; (**c**) HF-Removed: ECG signal after high-frequency (HF) noise removal; (**d**) ECG PLI-Removed: ECG signal after power-line interference (PLI) removal; (**e**) Normalized or preprocessed ECG signal.

Firstly, ECG segments were extracted from each record, focusing specifically on 10 s segments immediately following or preceding the collection of a corresponding blood gas sample collection event. This approach involved extracting segments from both the left and right sides of the blood gas sampling event. The rationale behind using these segments is that the maximum correlation between blood gas samples and ECG signals can be expected in segments that are temporally closest to the blood sampling event. This resulted in a dataset of 274 ECG segments with corresponding pH values. A representative two-second ECG segment from the dataset is displayed in Figure 1, along with the preprocessing steps applied to the data.

Next, each raw ECG segment was preprocessed before extracting ECG features to reduce the effect of noise. ECG recordings are typically contaminated with artifacts from different sources of noise. Noisy data may be attributed to infant activity and the small chest diameter relative to electrode size [54]. The most prominent artifacts include baseline wander, power-line interference (PLI), muscle artifacts, and electrode motion artifacts. These artifacts occupy low-frequency, medium-frequency, and high-frequency bands in ECG signals [55,56]. The preprocessing stage employed standard ECG filtering methods [57,58,59] to reduce the various types of noise present in raw ECG signals (Figure 1a). The first step was to remove the baseline drift using a high-pass Butterworth filter with a cut-off frequency of 0.5 Hz. This filter effectively removes any low-frequency noise that could have interfered with the ECG signal (Figure 1b). Next, a low-pass Butterworth filter with a cut-off frequency of 100 Hz was applied to remove the high-frequency noise including electromyographic noise [59]. This filter was used to eliminate any high-frequency noise that could have been introduced during the signal acquisition (Figure 1c) [60]. Power-line interference is a common problem in ECG signal acquisition and can cause significant artifacts in the signal [48]. The power-line interference was effectively eliminated by applying a notch filter at 50 Hz [45] (Figure 1d). After the initial preprocessing steps, the Savitzky–Golay filter was employed to improve the overall signal-to-noise. This filter effectively smooths the signal and eliminates any residual noise or artifacts that may have been present [58]. Unlike conventional filters such as the median and smooth filters, the Savitzky–Golay filter can effectively remove noise while preserving important signal features, which is crucial for accurate ECG analysis and feature extraction, particularly for the T-wave [47]. Following the application of the Savitzky–Golay filter, signal normalization was performed, as illustrated in Figure 1e. Figure 1 shows all the preprocessing steps of the ECG signals. While the preprocessing of the raw signals significantly reduced the noise, feature extraction algorithms were employed to extract the features from the relevant frequency band of the ECG signal.

**Figure 2:**
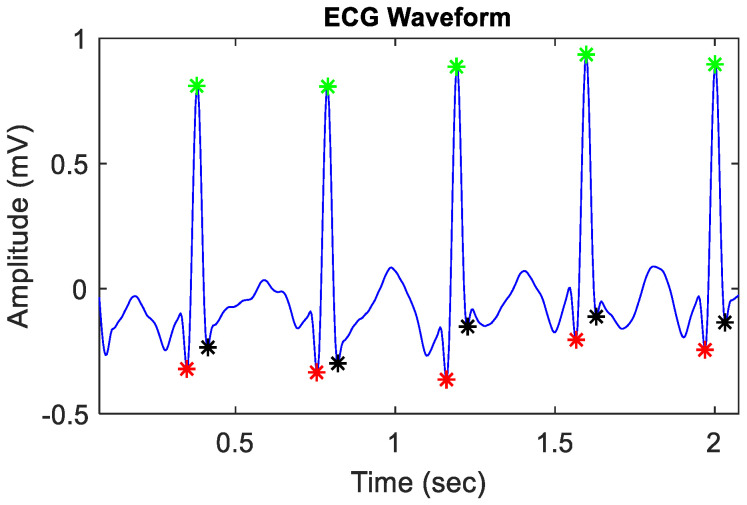
QRS-peak detection. On the ECG segment displayed in blue color, the red asterisk (⁕) represents the detected Q-wave peak position, the green asterisk (⁕) denotes the R-wave peak position, and the black asterisk (⁕) indicates the S-wave peak position.

Next, in the feature extraction process, QRS complex detection was performed before the calculation of T-wave feature extraction. The QRS detection algorithm is based on the work of Hamilton and Tompkins [49,50] and is designed to detect the QRS complex [50,56] (Figure 2). A nonlinear transform was applied to the filtered signal to further emphasize the QRS complexes. Adaptive thresholding was used to detect the QRS complexes based on the nonlinear transformed signal. Within each detected QRS complex, the algorithm locates the Q, R, and S points [61]. Specifically, the Q-peak is identified as the minimum value in the signal between the QRS onset and the R-peak. The R-peak is detected as the maximum value within the QRS complex. Furthermore, the S-peak is determined as the minimum value in the signal between the R-peak and the QRS offset [61]. The location of R-peaks was used to calculate RR-average and average heart rate (HR). If an ECG segment was too noisy and had missing ECG cycles, the QRS detection failed at this stage, and the ECG segment was discarded. Out of the 274 extracted segments, some contained missing ECG cycles, while others were excessively noisy, leading to a suboptimal performance of the PT algorithm. As a result, only 108 segments out of the 274 were selected for further evaluation.

Following the application of the PT algorithm, a representative ECG cycle was extracted from each segment, and period normalization was performed. After period normalization, principal component analysis (PCA) was employed to extract the most relevant ECG cycle for each segment. PCA is a powerful dimensionality reduction technique that can identify the most relevant principal components in the data while preserving as much of the original variability and physiological information as possible [53,62]. The normalized ECG segment was represented as an M × N matrix, where M denotes the number of samples within the segment, and N represents the number of ECG cycles, which served as the basis for calculating the covariance matrix. The eigenvectors and eigenvalues of the covariance matrix were then computed, and the principal components accounting for most of the variance were retained. This ECG cycle, obtained through the PCA-based dimensionality reduction technique, was then utilized for T-wave feature extraction and analysis [52,63]. The methodology included calculating RR intervals, defining windows around R-peaks for T-wave analysis, and interpolating the signal within these windows for standardization. T-wave signals and T-wave peak locations were reidentified using the PT algorithm. A windowing technique was employed to evaluate the T-wave shape, and the slope of the T-wave signal was determined. ECG features related to the T-wave, such as T-amplitude and T-slope, were computed. Following the identification of the QRS complex and T-wave features, the ECG features utilized in this study were computed. These features include T/QRS, T Amplitude, Tslope, Tslope/T, Tslope/|T|, HR, QT, QTc, RRAvg, and Tslope/sqrt|T|, as detailed in Table 1. The QTc interval was calculated using Bazett’s formula where $QTc=\frac{QT}{\sqrt{RR}}$ [64].

**Figure 3:**
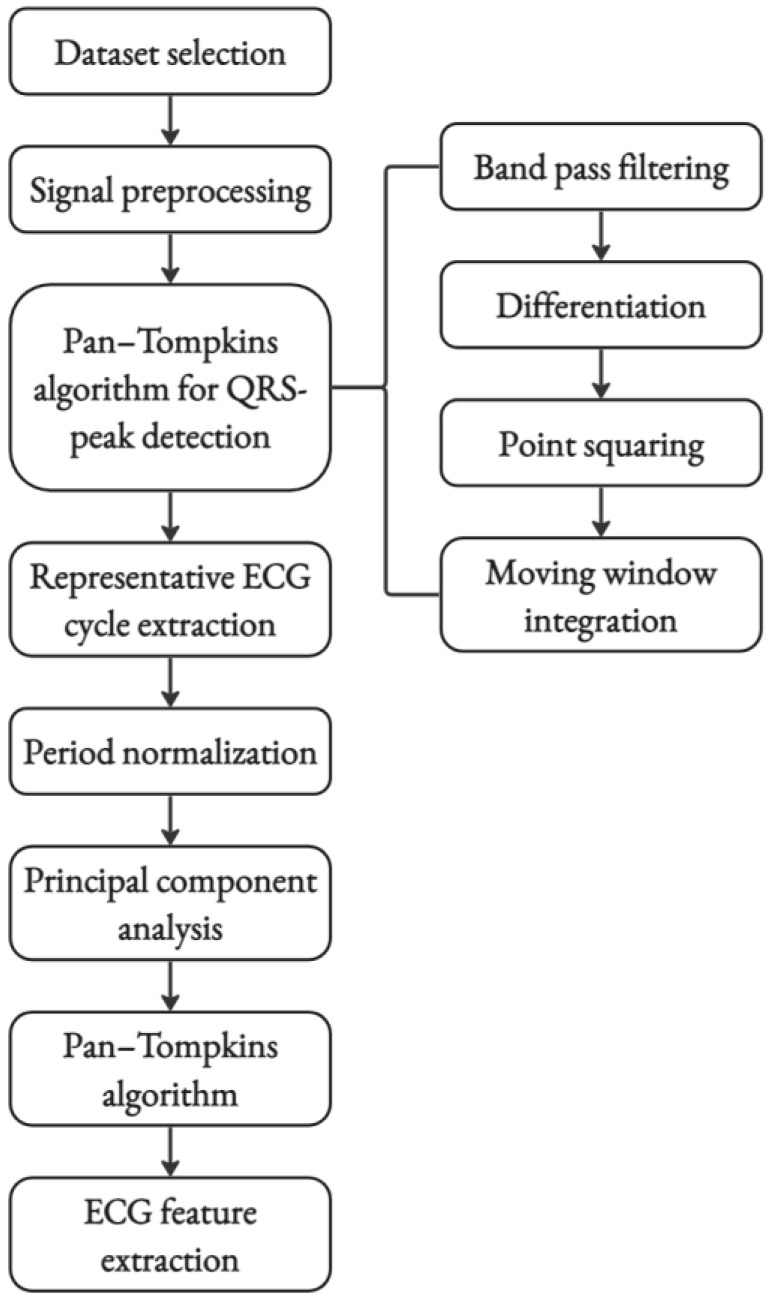
Flow chart of the ECG feature extraction used in this work. The process resulted in the features to be used for ECG statistical analysis.

The complete flow sheet diagram of the ECG feature extraction process is shown in Figure 3. The T-wave features may provide useful information about metabolic disorders [65], so T-wave features have been given more emphasis to evaluate metabolic disorders in asphyxiated neonates.

### 2.4. Statistical Analysis of ECG Features

The first step in assessing the acid-base imbalance is to evaluate the pH from the blood gas analysis. The acid-base imbalance gives information about acidosis and alkalosis disorders based on neonatal blood gas analysis [66]. Moreover, based on the pH value, the data is further categorized into three groups: 9 segments occur where the pH value is less than 7.20 (metabolic acidosis or acidosis), 83 segments occur where the pH ranges between 7.20 and 7.45 (this range is considered normal), and 16 occur segments where the pH is greater than 7.45 (metabolic alkalosis or alkalosis). Using these pH threshold values, in this study, neonatal condition was categorized into three groups: acidosis, normal, and alkalosis.

#### 2.4.1. Descriptive Analysis

Descriptive statistics were conducted on the ECG features using pH values in MATLAB R2023b (The MathWorks, Natick, MA, USA) to summarize the basic features of the data used in this study. The analysis conducted in this study utilized several MATLAB toolboxes and functions. Specifically, the Statistics and Machine Learning Toolbox was employed for regression analysis. The Curve Fitting Toolbox was used for smoothing operations. Additionally, the Signal Processing Toolbox was required for the application of the Butterworth filter. For graphical representation, box and whisker plots are used for the summarization and distribution of data between groups [67].

#### 2.4.2. Statistical Analysis

Statistical analysis using a nonparametric test was performed on MATLAB. Data measurements were expressed as the median [interquartile range (IQR)] to compare the groups. Given a small and imbalanced dataset, a multiple-group comparison test, the Kruskal–Wallis test, was used to analyze the differences between groups [68,69]. A post hoc test was performed after the multiple-group comparison test, and statistically significant differences were found based on the *p*-value. The Dunn–Šidák post hoc test was used to validate the Kruskal–Wallis test for comparing measurement data between the groups [68]. Hence, to keep the rank sums from the chi-square test after a Kruskal–Wallis test, the Dunn–Šidák post hoc test is the suitable approach. In the Dunn–Šidák post hoc test, the mean ranks of the two groups are significantly different if their intervals are disjointed; they are not significantly different if their intervals overlap. The *p*-value (*p* < 0.05) was treated as statistically significant [70].

## 3. Results and Discussion

**Figure 4:**
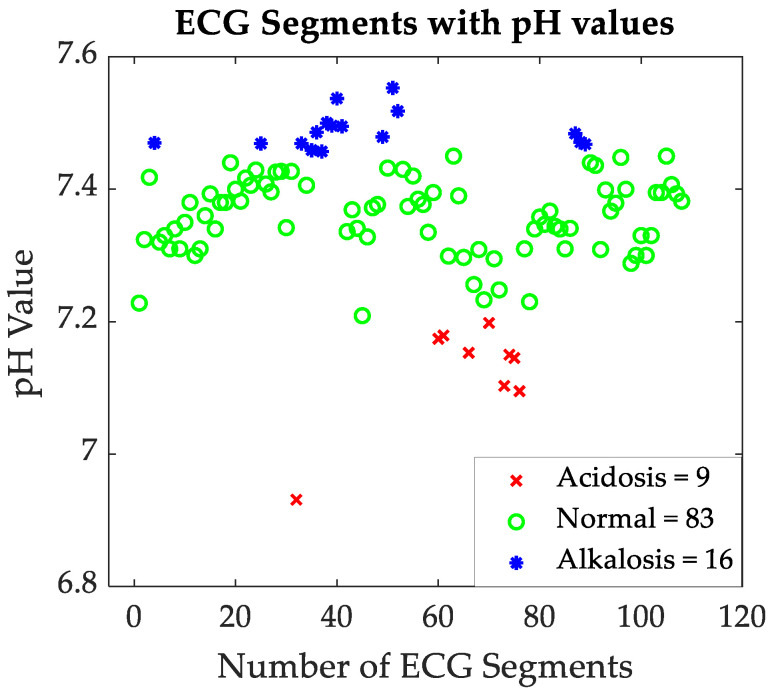
ECG segments vs. pH value.

The dataset used in our study has the data points of 49 patients with 274 segments where ECG waveform and pH values are recorded simultaneously. The data are further limited by the fact that only 9 segments exist in the dataset where the pH value is less than 7.20 (metabolic acidosis), 83 segments where the pH ranges between 7.20 and 7.45 (this range is considered normal), and only 16 segments where the pH is greater than 7.45 (metabolic alkalosis) (Figure 4). All ECG features used in this study are grouped (acidosis, normal, and alkalosis) by pH values.

### 3.1. Results from the Descriptive Analysis

**Figure 5:**
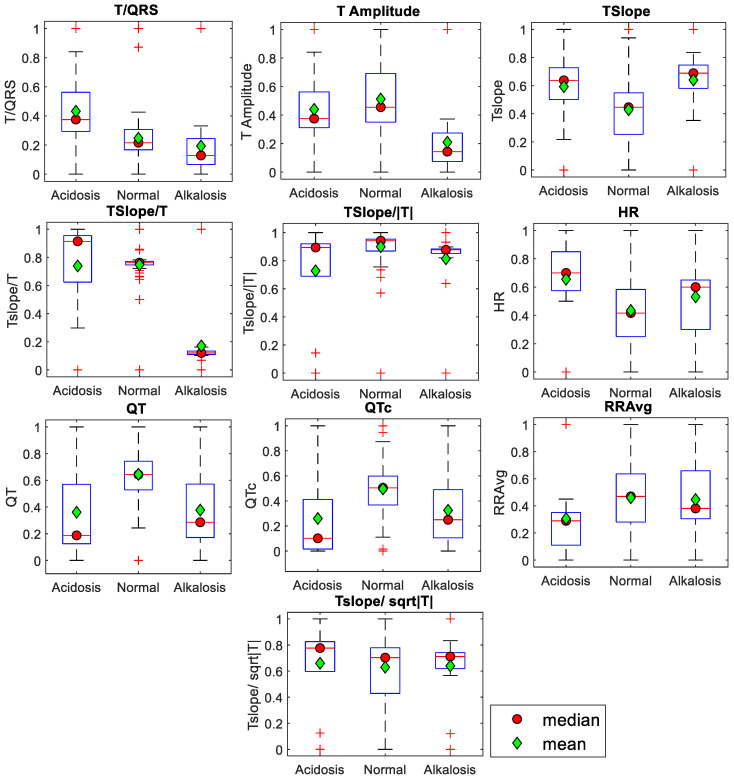
Descriptive analysis using box plots for the neonate’s group (acidosis, normal, alkalosis) represents the mean values (green diamond-shaped markers) and the median values (red dots) of the ECG features. These box plots and whisker plots illustrate that the data are not symmetrical, and the mean values are not equal to the median values around the median, suggesting that the data are not normalized.

The distribution pattern of the data (parametric or nonparametric) was observed by examining the results of the box and whisker plots. All three groups (acidosis, normal, and alkalosis) are displayed horizontally while the average values of each ECG feature are displayed vertically in Figure 5. The ECG feature values are scaled between 0 and 1 to obtain symmetric plots. The boxplots suggest that the data are not normal. Therefore, the nonparametric test was selected for further statistical analysis. To describe the main properties of the data utilized in this study, descriptive statistics were performed on the ECG features using pH values. The Kruskal–Wallis nonparametric test was used to analyze differences between the two groups, followed by the Dunn–Šidák post hoc test for individual comparison among the mean ranks of all groups.

### 3.2. Results from the Statistical Analysis

The Kruskal–Wallis test was performed to determine whether differences between groups exist. The output of the Kruskal–Wallis test in our dataset, for example, for T/QRS gives a *p*-value of 0.0073. Importantly, the chi-square is less than the calculated value of the Kruskal–Wallis test, indicating that at least one of the groups is different from the others. The results for other features including the T Amplitude, Tslope, Tslope/T, Tslope/|T|, HR, QT, and QTc are also significant, indicating that at least one of the groups in all these features is significantly different from the others. Two features (RRAvg and Tslope/sqrt|T|) as the output values for the Kruskal–Wallis test indicate that none of the groups is significantly different from the other groups. The statistical outputs are shown in Table 1.

If the null hypothesis stating that the ECG feature medians are the same across all groups is rejected, it becomes necessary to identify the specific locations where the differences among the medians exist. To determine which pairs of the medians are significantly different, and which are not, the Dunn–Šidák post hoc test was used. Pairwise comparisons using Dunn’s test for T/QRS indicated that the acidosis group’s (group 1) mean ranks were observed to be significantly different (*p* = 0.0064) from those of the alkalosis group (group 3). Similarly, for T Amplitude, the normal group’s (group 2) mean ranks are significantly different from the alkalosis group (group 3) (*p* = 0.0000). No other differences were statistically significant for the T/QRS and T Amplitude features. Similarly, the results for HR indicated that the acidosis group’s (group 1) mean ranks differed significantly from the normal group (group 2) (*p* = 0.0289), and no other differences were statistically significant. The statistical outputs are shown in Table 2.

**Figure 6:**
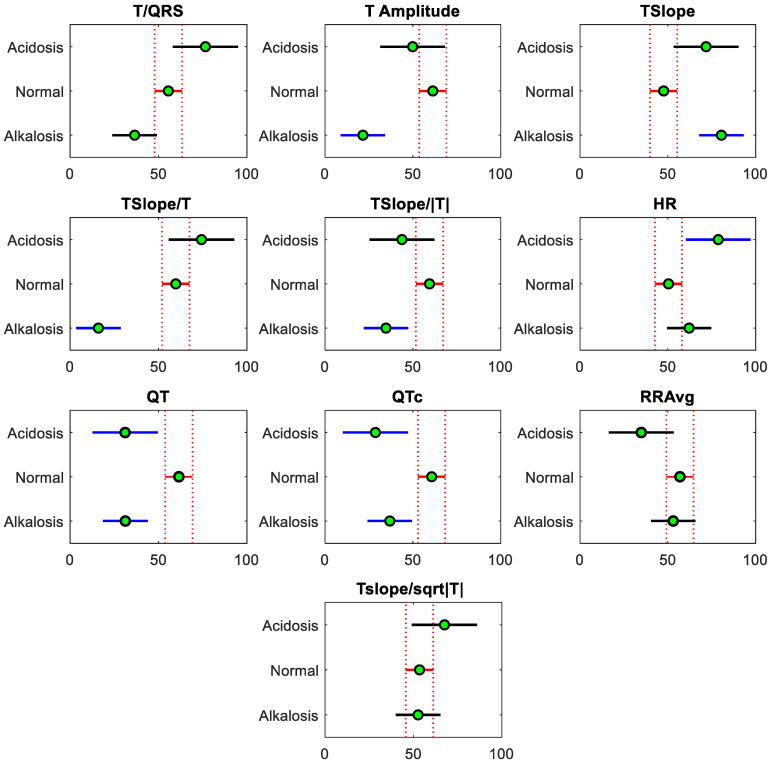
Interactive graphs illustrate the pairwise comparison of the estimates and comparison intervals to display the significant difference among all three groups (acidosis, normal, and alkalosis). The group means are along the x-axis, and the groups are categorized along the y-axis. The red intervals and estimates depict that the selected group is significantly different from others (blue intervals). Groups that do not have significantly different means to the normal group are displayed in black color.

The interactive graphs, in Figure 6, illustrate the pairwise comparisons for ECG features by visually representing the comparison intervals for the group means. For example, for ECG feature T Amplitude, the comparison intervals for the normal and alkalosis groups do not overlap; this indicates that the difference between the group means is statistically significant at the specified significance level (*p* < 0.05). Additionally, the interactive graph for HR shows that the comparison interval for the acidosis and normal group do not intersect. This lack of intersection suggests a significant difference between the means of these two groups. These significant differences in metabolic disorders in asphyxiated neonates compared to the normal group were also investigated in Table 2. If the neonate is experiencing a hypoxic condition leading to asphyxia, the results have shown that proper clinical management results in the normal condition of the neonate. However, poor management may result in metabolic disorders that can be assessed by pH value, using invasive blood sampling collection. Significant differences were evident between the normal condition of the neonate and metabolic disorders when ECG analysis was performed. Tslope/T, QT, and QTc are the main ECG features that are significantly different in all three groups (acidosis, normal, and alkalosis), as presented in Table 2. The ECG features T/QRS, T Amplitude, Tslope, Tslope/|T|, and HR are also key features that are significantly different from either of the two groups. RRAvg and Tslope/sqrt|T| are not significantly different in either group. The results presented in this study of neonatal ECG waveform analysis may help in a clinical decision on whether a blood sample is necessary during asphyxia management, to reduce the unnecessary blood sampling in asphyxiated neonates. While there are no previous studies on the relationship between neonatal blood pH and ECG features, the findings of this study are like those of previous studies observed in ECG-based fetal hypoxia detection. Most of the previous studies found ECG features such as T/QRS ratio, T-waveform features [34], QTc [71], and FHR [36] to be useful in fetal hypoxia detection. The results from this study validate previous results in fetal hypoxia detection studies [37,72] and support the employment of ECG-based hypoxia detection in the NICU. However, further investigations are needed to validate these preliminary results in a larger dataset as this study is limited by a small dataset of neonates recorded in a single center. In addition to the morphological features of ECG, the risk of fetal hypoxia and cardiac arrhythmias may be associated with the variation in HR and HRV features. However, in preterm infants (born before 37 weeks of gestation), HR and HRV features are also significantly correlated with patient data, such as birth weight and gestational age [73,74], which have not been analyzed in this preliminary investigation. While HR has been considered in analysis in addition to other morphological ECG features, HRV is excluded due to various factors, including neonatal body temperature during hypothermia (asphyxia management), influencing HRV [75].

Although ECG is the primary method to identify, monitor, and track the real-time onset, progression, and results of myocardial injury [24], studies suggest that serious myocardial damage may occur when diagnosed because ECG abnormities can only be detected after myocardial tissues and cardiac functions are impaired [31]. The delay between the onset of the asphyxia-related event and its detection in the ECG signal may be a concern in ECG-based asphyxia monitoring. While this is a concern for real-time detection, ECG-based continuous monitoring may still be beneficial over intermittent blood sampling methods. Moreover, in addition to the limited size of the study population, three subgroups have a different number of data points, which limits the ability to identify rare, serious, adverse events. The imbalanced and small-sized study also limits the tests applied for the statistical analysis as well as a robust regressor. Another main challenge during analysis is the poor quality of the ECG recordings, which may be interfered with by neonatal motion artifacts and variations in electrode placement [54]. These low- and high-frequency noises may have an impact on signal preprocessing, QRS-peak detection, ECG feature extraction, and the further analysis of ECG features.

This study utilized a unique dataset that includes both physiological-based (ECG) and chemical-based (ABG) biomarkers recorded concurrently. Due to the limitations of the dataset, such as its small size and imbalance, statistical analysis was performed to identify ECG morphological features significantly different based on pH values, which indicate whether the conditions of the asphyxiated neonate is normal, acidotic, or alkalotic. Additionally, in this study, only one parameter (pH value) was considered for acid-base characterization. However, other parameters including base excess (BE), lactate, partial pressure of O_2_ (pO_2_), partial pressure of CO_2_ (pCO_2_), and bicarbonate (HCO_3_^−^) can play an important role in determining the metabolic disorders in neonates [22,76]. Due to the unavailability of complete demographic information, the statistical analysis for demographic variables such as gender, gestational age, birth weight, and mode of delivery was not performed in this study. However, the introduction of a large and comprehensive birth asphyxia dataset with complete demographic information can be further explored to establish correlations with pH-based ECG morphological features and patient demographic data. Future studies should consider these factors in larger cohorts using statistical analysis with advanced machine learning and deep learning algorithms to extend these results toward the noninvasive and accurate monitoring of asphyxia.

The reliable detection of asphyxia using ECG features is challenged by the probability of false negative and false positive results, as well as the overlap in statistical parameters between asphyxiated and non-asphyxiated cases. Limitations in feature extraction techniques, individual variability in ECG responses, and the presence of noise and artifacts can contribute to false negatives. Conversely, the partial overlap in T-wave characteristics and other ECG features between the two conditions makes it difficult to establish clear decision boundaries, leading to false positives. To address these challenges, a comprehensive approach is required, involving the exploration of additional ECG features, the integration of multicenter data sources, and the development of robust classification algorithms. By adopting this approach, the accuracy and reliability of ECG-based asphyxia detection can be improved, reducing the probability of both false negative and false positive results.

This study may aid clinicians in interpreting metabolic acidosis and birth asphyxia. However, a larger cohort dataset is necessary to formulate a hypothesis to classify metabolic disorders and predict birth asphyxia noninvasively based on these features. An extended similar retrospective dataset in the context of asphyxiated patients with parameters measured from blood sampling and optimized ECG-feature-processing algorithms will enable the implementation of advanced machine learning or deep learning techniques. For instance, convolutional neural networks (CNNs) or recurrent neural networks (RNNs) could be trained using an extended dataset to learn the complex patterns and relationships between ECG signals and pH values. This approach might enable the development of more accurate and robust models for predicting pH levels from ECG data, which could have important implications for clinical predictions, assisting clinicians in making accurate and timely decisions for the effective management of birth asphyxia.

## 4. Conclusions

This paper presents the first evaluation of the behavior of ECG features in asphyxiated neonates while measuring the blood gas analysis simultaneously. This study showed that ECG features (T/QRS, T Amplitude, Tslope, Tslope/T, Tslope/|T|, HR, QT, and QTc) significantly differ in neonates with asphyxia compared to non-asphyxiated neonates. Additionally, Tslope/T, QT, and QTc are the main ECG features that are significantly different in all three groups (acidosis, normal, and alkalosis), before and after the management of asphyxia in neonates. Therefore, using all these significant ECG features or a combination of these, this study has investigated the feasibility of noninvasive, accurate, and continuous monitoring techniques for asphyxiated neonates. Although this study has used a small and imbalanced dataset to measure ECG features that are the important markers to predict the neonatal condition after birth asphyxia, our study may guide clinicians to consider the significant ECG features in the clinical management of asphyxiated neonates. Further studies in larger cohorts will extend these results toward the noninvasive and accurate monitoring of asphyxia.

## Figures and Tables

**Table 1:** A Kruskal–Wallis test was performed to determine the difference between groups based on the chi-square value and *p*-value < 0.05.

ECG Features	Chi-Square Value	*p*-Value
T/QRS	9.85	0.0073
T Amplitude	21.86	0.0000
T slope	17.78	0.0001
Tslope/T	30.14	0.0000
Tslope/|T|	9.56	0.0084
HR	7.80	0.0202
QT	18.02	0.0001
QTc	14.44	0.0007
RRAvg	4.05	0.1319
Tslope/√|T|	1.72	0.4242

**Table 2:** A pairwise comparison of ECG features was performed to show a significant difference between groups using the Dunn–Šidák post hoc test.

ECG Features	Groups		Mean Ranks	Interval		*p*-Value
T/QRS	1 *	3 ***	8.8955	40.0556	71.2156	0.0064
T Amplitude	2 **	3	19.3682	39.7869	60.2056	0.0000
Tslope	2	3	−53.3043	−32.8855	−12.4668	0.0004
Tslope/T	1	3	27.1733	58.3333	89.4934	0.0000
	2	3	23.3524	43.7711	64.1898	0.0000
Tslope/|T|	2	3	4.3757	24.7944	45.2131	0.0112
HR	1	2	2.1611	28.3186	54.4761	0.0289
QT	1	2	−56.6424	−30.4070	−4.1715	0.0169
	2	3	9.8567	30.2681	50.6795	0.0012
QTc	1	2	−58.2769	−32.0321	−5.7873	0.0107
	2	3	3.4051	23.8238	44.2425	0.0159

* Alkalosis group, ** Normal group, *** Acidosis group.

## Data Availability

The data that support the findings of this study are available on request from the corresponding author. The data are not publicly available due to privacy and ethical restrictions.
